# Increased prediction of right nonrecurrent laryngeal nerve in thyroid surgery using preoperative computed tomography with intraoperative neuromonitoring identification

**DOI:** 10.1186/1477-7819-12-262

**Published:** 2014-08-20

**Authors:** Er-li Gao, Xian Zou, Ye-hui Zhou, Dao-hai Xie, Jin Lan, Hong-geng Guan

**Affiliations:** Department of General Surgery, The First Affiliated Hospital of Soochow University, No. 188 Shizi Street, Suzhou, 215006 Jiangsu People’s Republic of China; Department of General Surgery, Jiangyuan Hospital Affiliated to Jiangsu Institution of Nuclear Medicine (Key Laboratory of Nuclear Medicine, Ministry of Health/Jiangsu Key Laboratory of Molecular Nuclear Medicine), 20 Qianrong Rd, Wuxi, 214063 Jiangsu People’s Republic of China; Department of Radiology, The First Affiliated Hospital of Soochow University, No. 188 Shizi Street, Suzhou, 215006 Jiangsu People’s Republic of China

**Keywords:** nonrecurrent laryngeal nerve, thyroid surgery, CT, IONM

## Abstract

**Background:**

A nonrecurrent laryngeal nerve (NRLN) is a rare but potentially serious anatomical variant. Although the incidence is reported to be 0.3% to 1.3%, it carries a much higher risk of palsy during thyroid surgery. The objective of this study is to investigate the usefulness of computed tomography (CT) for preoperative identification and intraoperative neuromonitoring identification (IONM) of NRLN in thyroid cancer patients.

**Methods:**

The preoperative neck CT scans from 1,574 patients who needed thyroid surgery were examined. Absence of the brachiocephalic artery (BCA) and the presence of *arteria lusoria* were defined as positive with NRLN. Systematic intraoperative neuromonitoring (IONM) was also carried out for these 1,574 patients to localize and identify NRLN. A negative electromyography (EMG) response from lower vagal stimulation but a positive EMG response from the upper position indicated the occurrence of an NRLN.

**Results:**

Nine NRLN (0.57%) were intraoperatively identified out of the 1,574 patients, and no patient with a NRLN showed preoperative clinical symptoms related to NRLN. Prior to the operation, surgeons identified only seven suspected NRLN cases based on identification of *arteria lusoria*. But a review of CT scans revealed that all cases could be identified by vascular anomalies. All patients were successfully detected at an early stage of operation using intraoperative neuromonitoring (IONM). Postoperative vocal cord function was normal in all patients.

**Conclusions:**

CT of the neck is a reliable method for predicting NRLN before thyroid cancer surgery. However, some image features can be easily missed. Neurophysiology helps the surgeon to identify the NRLNs more precisely. Combining the two evaluation methods may decrease the incidence of nerve palsy, especially in cases of NRLN. Considering that CT is expensive, requires an X-ray, and achieves less information than ultrasound (US) concerning thyroid nodules, we suggest that applying US and IONM is more reasonable.

## Background

The nonrecurrent inferior laryngeal nerve (NRLN) is a rare anatomical variant. In 1823, Stedman first reported a case of NRLN [1]. It almost always exists on the right side (0.3% to 1.6%), while the left NRLN is even rarer, appears to have an incidence rate of approximately 0.04% and is always associated with *situs inversus* [2–5]. Although the incidence is extremely low, NRLN is vulnerable to damage during thyroid cancer surgery, with resultant vocal cord paralysis [2, 4, 6]. The reported incidence of nerve injury during surgery in cases of NRLN is nearly 12.9%, while in the recurrent laryngeal nerve (RLN) it is 1.8% [4]. Hence, preservation of the nonrecurrent inferior laryngeal nerve intraoperatively is an extremely challenging procedure, even to experienced surgeons. To avoid nerve injury, a modification of standard thyroid surgery techniques is not only required, but preoperative identification of NRLN is even more crucial. Computed tomography (CT) scans of the neck have been widely applied as a routine preoperative evaluation for head and neck surgery. On the other side, in order to predict inadvertent nerve injury, intraoperative neuromonitoring (IONM) has commonly been applied in thyroid cancer operation to localize and identify RLN, but the usefulness of IONM for detecting NRLN has been described by only a few studies [5, 7, 8]. Although some imaging characteristics of preoperative neck CT suggest the presence of an NRLN, it can also occur without a subclavian artery anomaly or even occur on the left side [2, 9, 10]. Furthermore, there is no research describing the combined usage of the two techniques. The aim of this study is to assess the value of preoperative neck CT examination and IONM and to investigate whether the two evaluation procedures may assist diagnosis of NRLN.

## Methods

From September 2008 to December 2012, a consecutive 1,574 patients with thyroid nodule required surgical treatment in the Department of Surgery at the Institute of Thyroid Disease, Wuxi, China. This study was reviewed and approved by the hospital ethical committee and required informed consent from each patient for use of individual data profiles. Preoperative CT of the neck and intraoperative neuromonitoring (IONM) was routinely performed on all patients. Preoperative and postoperative vocal cord function was assessed by laryngoscope in every patient in the study, focusing particularly on those with NRLN.

**Figure 1:**
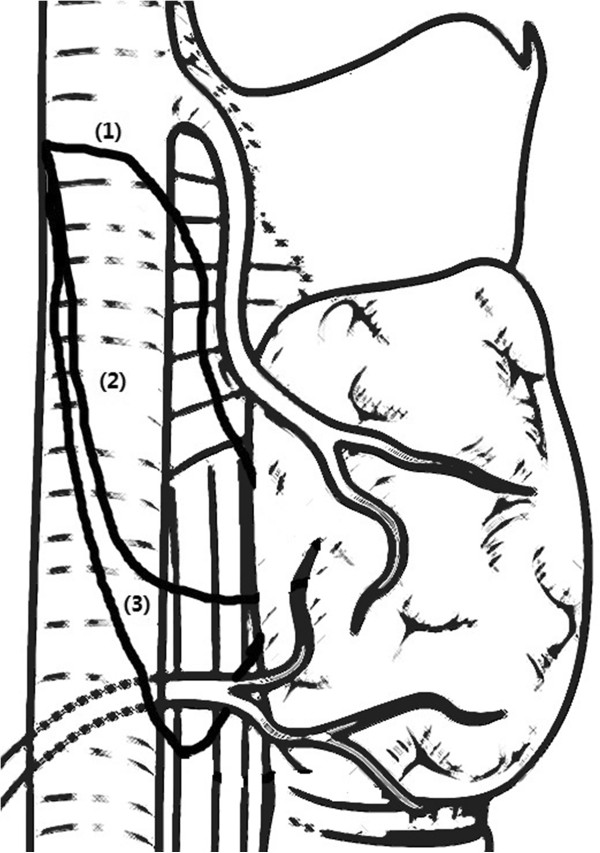
**Three types of nonrecurrent laryngeal nerve (NRLN) as described in the literature and based primarily on the course the nerve travels.**

To determine whether NRLN could be identified preoperatively, CT scanning images were reviewed retrospectively by the expert radiologist (Dr. Xie) in this study. An *arteria lusoria* was identified on CT scans as a tubular structure that arose from the dorsal side of the aortic arch. It passed through the midline behind the trachea and esophagus, entered the right base of the neck and proceeded as a right subclavian artery that joined the right common carotid artery. Based on the positional relationship between the NRLN and thyroid artery observed by the surgeons intraoperatively, the NRLN was classified into three types (Figure 1), as described by Toniato *et al*. [4].

All patients underwent thyroid cancer operations with the application of IONM. An electromyography (EMG) signal was recorded on an NIM-response 2.0 monitor (Medtronic Xomed). The four-step procedure of IONM (V1/R1/V2/R2) was systematically performed as recommended by Chiang *et al*. [11]. The original EMG signal from the vagus nerve was routinely tested by touching it directly with the nerve stimulator. The vagus was also stimulated at the level of the inferior thyroid pole with a current of 2 mA. Negative EMG signals at a lower position and positive EMG signals at an upper position indicated the occurrence of an NRLN, especially for those cases suspected on preoperative CT scan. The separation point and path of NRLN were localized and identified precisely. The thyroid gland was not removed until the NRLN was dissected.

## Results

Nine NRLN cases were identified out of 1,574 patients, and all were right-sided. Table 1 summarizes the clinical characteristic of the patients. There were only seven cases recognized in the CT images by the surgeons and radiologist. However, we reviewed the CT scans of all nine patients, and the other two cases were also identified retrospectively by the expert radiologist based on the absence of the right brachiocephalic trunk. Further observation shows that eight cases are type 1 and only one case is type 2A.

**Table 1:** **Clinical characteristics of nine nonrecurrent laryngeal nerve (NRLN) patients**

No.	Sex	Age (y)	Type of operation	Pathology
1	Male	40	Thyroidectomy + right-central cervical lymph node dissection	Papillary carcinoma on the right side
2	Male	59	Total thyroidectomy	Papillary microcarcinoma on the right side
3	Male	59	Right thyroidectomy	Nodular goiter
4	Female	71	Thyroidectomy + left-central cervical lymph node dissection	Papillary carcinoma on the left side
5	Female	55	Total thyroidectomy	Papillary microcarcinoma on the right side
6	Female	58	Thyroidectomy + bilateral central neck lymph node dissection	Papillary carcinoma at the isthmus
7	Male	54	Total thyroidectomy	Papillary carcinoma on the left side
8	Male	60	Total thyroidectomy + left-central cervical lymph node dissection	Papillary carcinoma on the left side
9	Female	24	Right thyroidectomy	Nodular goiter

**Figure 2:**
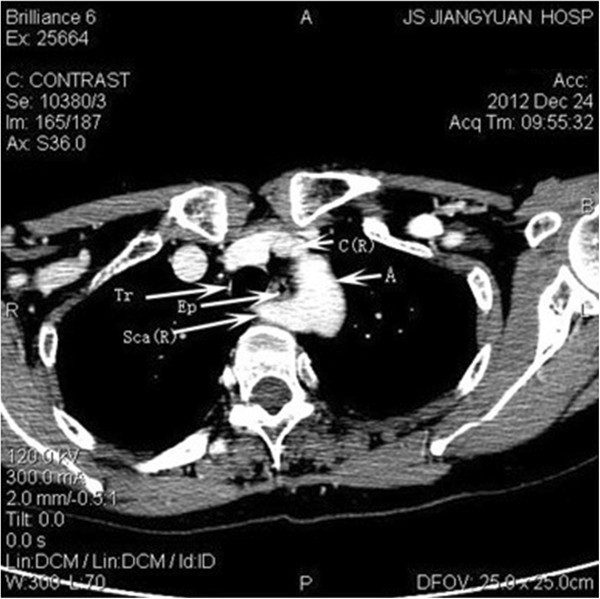
**Computed tomography (CT) scans showing the** ***arteria lusoria*** **coming from the dorsal side of the aortic arch.**

**Figure 3:**
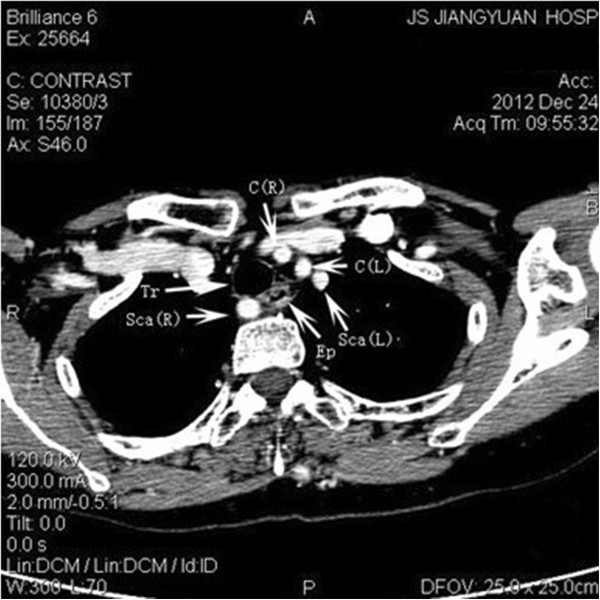
**The right subclavian artery can be observed behind the trachea and esophagus.**

**Figure 4:**
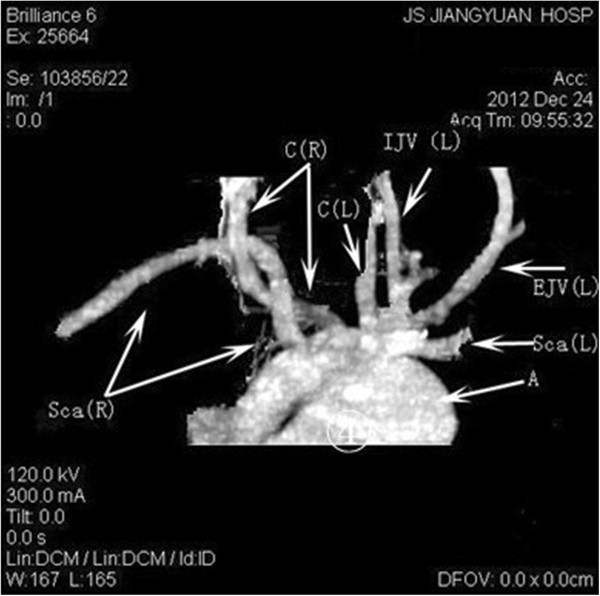
**Computed tomographic angiography showing the absence of the brachiocephalic artery; the right subclavian artery and right common carotid artery originate from the aortic arch.** Sca(L), left subclavian artery; Tr, trachea; Sca(R), right subclavian artery; C(R), right common carotid artery; C(L), left common carotid artery; A, aortic arch; Ep, esophagus.

All nine NRLNs (0.58%) were detected with IONM. Specifically, the two patients without preoperative recognition were also detected due to the negative EMG signals from the lower portion but positive responses from the upper portion vagal stimulation. None of the nine cases developed permanent or temporary palsy after surgery. There was not any complication attributed to the application of IONM. Computed tomographic angiography (CTA) was performed in five cases. The imaging showed that the *arteria lusoria* originated from the distal part of the aortic arch, traveled through the esophagus posteriorly and reached the right axillary area (Figures 2 and 3). Magnetic resonance angiographic (MRA) imaging also showed that the brachiocephalic artery was absent but that the *arteria lusoria* was present (Figure 4).

## Discussion

The anatomic variant of a nonrecurrent laryngeal nerve is extremely rare. The first observation of it was described by Stedman in 1823 [1]. It nearly always occurs on the right side, while the left NRLN is even rarer with an incidence of approximately only 0.004% and is always associated with dextrocardia or even more complex vascular anomalies [2, 4, 12]. In our study, the incidence of NRLN, as surgically confirmed, was 0.57% (9/1,574). In spite of the low incidence, the presence of NRLN has become a high-risk factor for nerve injury during surgery, especially for thyroid cancer surgery.

Nowadays, most experts believe that the occurrence of the NRLN results from an abnormality of aortic arches during the development of the early embryo. During the growth of the embryo and descent of the heart, the laryngeal nerve lies beneath the sixth aortic arch and ascends to the larynx. On the left side, the RLN wraps around the sixth aortic arch, which ultimately forms the ligamentum arteriosum; meanwhile, on the right side, the distal parts of the sixth aortic arch and the fifth aortic arch disappear, and the nerve moves upward to lie beneath the fourth aortic arch, which forms the initial part of the subclavian artery. If the right fourth aortic arch is absent, the right RLN is free to move upward, originating directly from the vagus nerve at a cervical level and entering the larynx transversely. That is why the NRLN is almost always observed on the right side as left-side cases require the coexistence of a right aortic arch associated with dextrocardia, a left subclavian artery with a lusoria course, and the absence of an arterial ligament on the left side [2]. The right subclavian artery is often formed from the distal portion of the right dorsal aorta and the seventh intersegmental artery, and originates just below the left subclavian artery to reach the right axillary area in most NRLN cases. The presence of this aberrant right subclavian artery was first named *arteria lusoria* by Stedman [1].

There are three types of NRLN described in literature, and these are based primarily on the traveling course of the nerve [4] (Figure 1). In type 1, the nerve arises directly from the cervical vagus and runs together with the superior thyroid pedicle. In type 2A, the nerve runs transversely over the trunk of the inferior thyroid artery. In type 2B, the nerve runs transversely under the trunk or between the branches of the inferior thyroid artery. Our study also used this typing method. The results observed show that the most common is type 1 (8/9) and only one case is type 2A, which is different from the study by Lee [3].

Because the NRLN is very vulnerable, we lack effective methods to make it 100% safe. Most cases are diagnosed intraoperatively, as they do not have characteristic clinical symptoms or signs. However, we should pay attention to the fact that the NRLN usually associates with a vascular anomaly. Hence, if we could identify such vascular anomalies before the operation, we can make a preoperative diagnosis and avoid nerve injury.

Various methods have been described to make a preoperative diagnosis, but each examination has its own limitation. Barium esophagogram is recommended as a part of the preoperative procedure before head and neck surgery in patients with dysphagia. The results of a barium swallow test usually reveal a notch on the left edge of the posterior esophagus. However, this sign can be caused by thyroid nodules and can also easily be missed if not carefully examined [13, 14]. Ultrasonography is a noninvasive, rapid and inexpensive method to evaluate thyroid diseases preoperatively. Unfortunately, a study of its efficacy in identification of an NRLN has not been evaluated. But more than one study has found that ultrasonography is a very reliable and simple method for identifying vascular anomalies associated with NRLN preoperatively [13, 15, 16]. MRI and endoscopic ultrasound are both reliable methods with high diagnostic accuracy. But whereas MRI is limited because of its high cost, endoscopic ultrasound is limited because of its invasiveness and high cost [14, 17, 18]. Angiography is one of the most effective and directive methods to diagnose vascular anomalies, but it is inappropriate to use before thyroid cancer surgery because the dye for angiography contains a lot of iodine, which may affect the postoperative radioactive iodine treatment [19].

Computed tomography of the neck is accepted as a routine procedure before thyroid cancer surgery in some medical centers. The presence of an *arteria lusoria* and the absence of the brachiocephalic artery can be seen directly and establish a diagnosis of NRLN [20, 21]. The result of the present study shows that it is possible to predict NRLN by identifying the presence of a vascular anomaly. Despite this, we identified missed diagnoses (2/9), probably because some radiologists do not expect to find it.

The occurrence of nerve injury of the NRLN in thyroid surgery is higher than RLN (12.9% versus 1.8%) [4]. The incidence may be higher when the surgeons are unfamiliar with the anatomical variation [22]. And most cases were confirmed during thyroid surgery. Thus, prevention of nerve injury requires not only an accurate preoperative diagnosis of the NRLN but also a method to localize its separation point and path precisely during the thyroid cancer surgery.

Although CT or US of the neck has the ability to predict the presence of the NRLN preoperatively, it cannot ensure absolutely the safety of the nerve during the surgery. Intraoperative neuromonitoring (IONM) has been accepted as a method to localize and identify the RLN and predict postoperative cord function [23, 24]. Recently, Donatini *et al*. demonstrated that systematic IONM revealed a higher incidence of NRLN than expected [5]. The present study also successfully detected another two NRLN cases that were misdiagnosed with CT scans before the surgery. When NRLN is suspected from the CT scan or ultrasound images, surgeons should pay more attention to measurements of the EMG signals from IONM and then dissect the vagus nerve. Once the EMG signal is absent from the lower position but positive at the upper position, we could consider the occurrence of an NRLN. Our experience demonstrates the following: (1) the position where the nerve penetrates the larynx is important to the anatomy no matter whether it is recurrent; (2) when we cannot find the RLN during the surgery, we should expect the occurrence of an NRLN; (3) preoperative CT scans could be performed, especially for those who are suspected thyroid cancer patients, but since CT is expensive, requires X-ray and achieve less information than US concerning thyroid nodules, we suggest that routinely using ultrasound should be more reasonable; and (4) routine application of IONM is advised to localize and identify the RLN and further predict the dysfunction of the vocal cord.

## Conclusions

In summary, when thyroid cancer is suspected, we recommend that imaging examination (US/CT) should be performed preoperatively, and IONM should be applied during the surgery. Combining the two reliable methods is effective in predicting vocal cord dysfunction, especially for those patients with NRLN.

## Authors’ information

Gao Erli is a surgeon in the Department of General Surgery, The First Affiliated Hospital of Soochow University, Suzhou, Jiangsu, People's Republic of China. Zou Xian is a surgeon in the Department of General Surgery, Jiangyuan Hospital Affiliated to Jiangsu Institution of Nuclear Medicine, Wuxi Institution of Thyroid disease, Wuxi, Jiangsu, People's Republic of China. Zhou Yehui is a surgeon in the Department of General Surgery, The First Affiliated Hospital of Soochow University, Suzhou, Jiangsu, People's Republic of China. Xie Daohai is a radiologist in the Department of Radiology, The First Affiliated Hospital of Soochow University, Suzhou, Jiangsu, People's Republic of China. Lan Jin is a surgeon in the Department of General Surgery, The First Affiliated Hospital of Soochow University, Suzhou, Jiangsu, People's Republic of China. Guan Honggeng is an Associate Chief Physician of the Department of General Surgery, The First Affiliated Hospital of Soochow University, Suzhou, Jiangsu, People’s Republic of China.

## Abbreviations

BCA: brachiocephalic artery
CT: computed tomography
CTA: computed tomographic angiography
EMG: electromyography
IONM: intraoperative neuromonitoring identification
MRA: magnetic resonance angiographic imaging
NRLN: nonrecurrent laryngeal nerve
RLN: recurrent laryngeal nerve.
